# Case report: Recurrence of psychosis after the surgical resection and radiation of a temporal lobe astrocytoma

**DOI:** 10.3389/fpsyt.2024.1485502

**Published:** 2025-01-06

**Authors:** David Perekopskiy, Shervin Zoghi, Jenna Dobrick, Orwa Aboud, James Alan Bourgeois

**Affiliations:** ^1^ Department of Psychiatry and Behavioral Sciences, University of California, Davis Medical Center, Sacramento, CA, United States; ^2^ Department of Neurological Surgery, University of California, Davis Medical Center, Sacramento, CA, United States; ^3^ Department of Neurology, University of California, Davis Medical Center, Sacramento, CA, United States; ^4^ Comprehensive Cancer Center, University of California, Davis Medical Center, Sacramento, CA, United States

**Keywords:** psychosis, neuroimaging, risperidone, amphetamine, tumor, astrocytoma, secondary psychotic disorders

## Abstract

It is estimated that the incidence of first episode psychotic disorder is about 33 people out of 100,000 each year. Beyond primary psychotic illness (e.g., schizophrenia, schizophreniform disorder), some of these patients will develop psychotic disorder due to a complex interplay of genetics, anatomical variations, traumatic brain injury (TBI), environment, substance use, and/or other causes. A small subset of patients will develop psychotic disorder due to a structural anatomic lesion, such as a CNS tumor. Here we present a 35-year-old male with worsening auditory hallucinations after surgical resection and radiation of a right temporal lobe astrocytoma in the setting of co-morbid methamphetamine usage. This case report helps illustrate how a neuroimaging work-up is important for the first incidence of psychotic disorder and how a tumor can produce a psychotic disorder that persists after oncologic treatment. This paper adds to the literature on the presentation and treatment of post-resection tumor-induced psychotic disorder.

## Introduction

A 35-year-old male presented to the emergency department due to concerns raised by his neurooncologist during a follow up appointment for worsening delusions and auditory hallucinations after surgical resection and radiation therapy of a right temporal anaplastic astrocytoma, WHO grade 3.

Prior to the detection of the tumor, the patient had experienced two years of gradually worsening psychotic symptoms. During this time, he had multiple emergency department visits due to psychotic presentations, including being brought in by law enforcement officers for breaking into a house, responding to internal stimuli, auditory hallucinations, and delusions about “Freemasons and The Illuminati.” His urine toxicology screen had been positive for methamphetamines during multiple ED visits. There was no record of long term antipsychotic treatment. Seizures subsequently started one year prior to the cancer diagnosis, although seizures were not reported in records of the emergency department visits.

**Figure 1:**
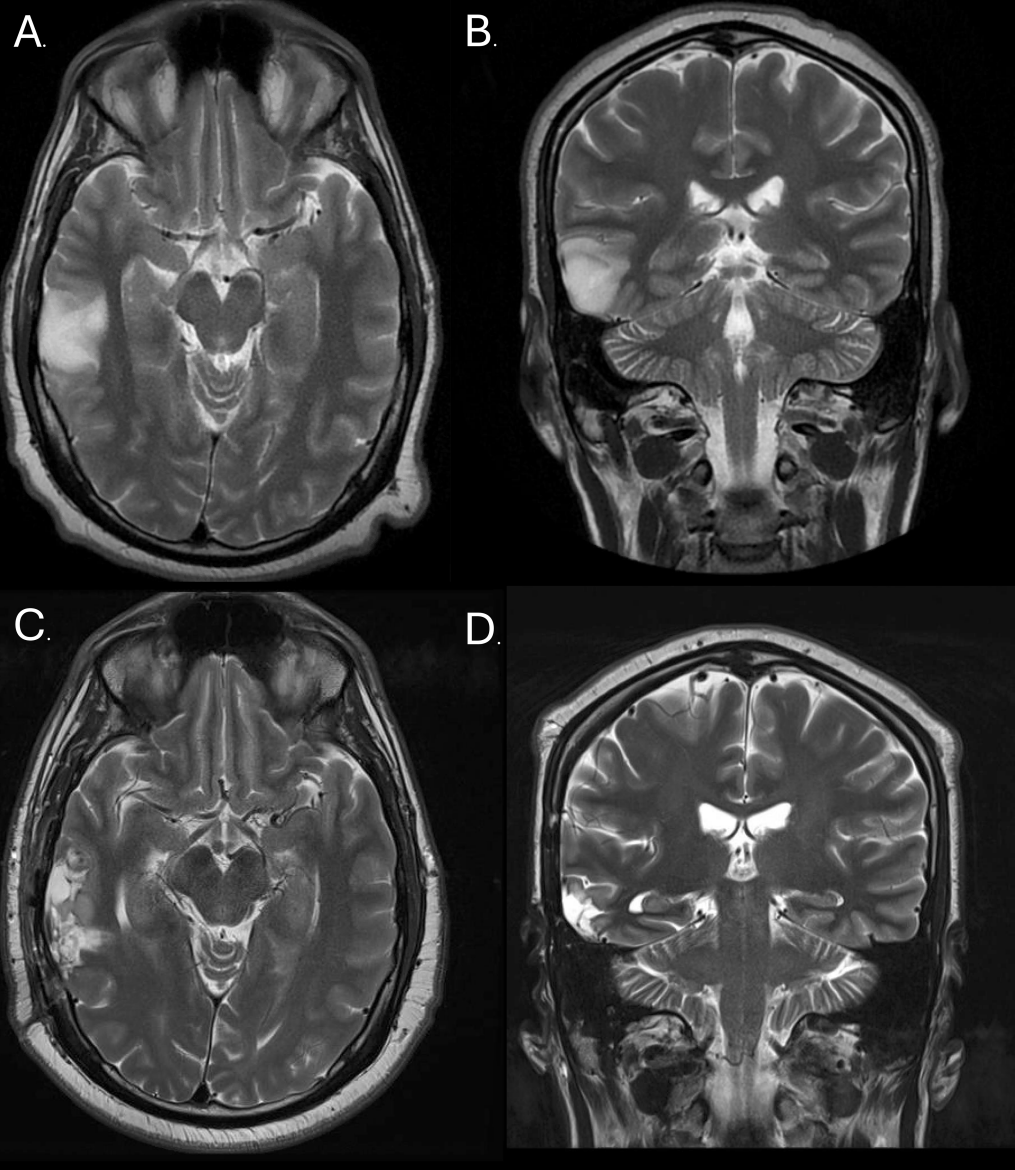
Right Temporal Astrocytoma: A T2-Weighted MRI image prior to resection (transverse **(A)** and frontal **(B)** planes) and post-resection (transverse **(C)** and frontal **(D)** planes).

Over a year after his first ED visit for psychotic disorder, the patient presented to the ED with a seizure; a CT and MRI were obtained. Neuroimaging revealed a tumor involving the right middle and inferior temporal lateral gyri, measuring 40 x 27 x 28 mm (**Figure 1**). This tumor, a right temporal anaplastic astrocytoma, WHO grade 3, IDH mutant, was surgically resected 10 months prior to the most recent presentation, followed by 33 treatments of radiation over 7 months. After completion of his initial oncologic therapy, the patient’s family reported that he had no further psychotic symptoms while maintained on levetiracetam 1000 mg twice a day. However, four months later, he developed recurrence of delusions and onset of erratic behavior.

Upon presentation to the ED from the neuro-oncology visit, he was alert, pleasant and well-groomed. He denied depressive or manic symptoms, but reported auditory hallucinations, ideas of reference, thought insertion, and believed that he had “super-powers” and “telepathy.” On mental status exam, he was alert, cooperative, normokinetic, and speaking with normal rate but soft, slightly dysarthric speech. His thought process was circumstantial to tangential. The Patient’s initial partial Montreal Cognitive Assessment (MoCA) was consistent with moderate cognitive impairment at 14/22. He was not tested on visuospatial/executive function and naming categories. The patient misidentified the date, day of the week and month (3/6). The patient was able to recall 3 out of 5 words (3/5), complete 1 serial 7s (1/3) and was not able to name the months of the year backwards (0/1). His abstraction and language were intact. The patient’s urine toxicology screen was positive for methamphetamine. Workup included MRI of the brain with and without contrast that showed stable findings without evidence to suggest tumor recurrence. EEG was performed and was negative for epileptiform discharges.

In the ED he was initially started on olanzapine 10 mg daily due to a prior positive response after his neurosurgery; after his surgery he had had cessation of his delusions and AH at 10 mg olanzapine daily. Patient was tapered off but was warned his symptoms might come back. In the ED he was later switched to risperidone 4 mg at bedtime to facilitate potential transition to a long acting injectable antipsychotic. The patient expressed decreased auditory hallucinations but continued describing delusions about “The Illuminati, angels, and clairvoyants.” On day 7, the patient had a legal certification hearing where his psychiatric commitment order for grave disability was upheld. The patient’s risperidone was up-titrated to 6 mg at bedtime due to continued, though attenuated, delusions affecting the patient’s ability to create a reality-based plan.

After 3 days on risperidone 6 mg at bedtime, he was then able to create a reality-based plan for obtaining food and shelter and he was no longer experiencing psychotic symptoms. After stabilization, the patient was able to explain that his persistent delusions about angels, The Illuminati, and other ideas had originated from vivid visual hallucinations during seizures prior to the discovery of his tumor. At the end of his hospitalization, the patient scored a 28/30 on the full MoCA, missing points for only for 4/5 recall and clock contour. The patient was discharged after 11 days of hospitalization.

## Discussion

Whenever a patient presents with new onset psychotic disorder, it is important to assess all possible secondary causes of psychotic illness (1) (**Table 1**). Temporal lobe tumors are rare malignancies that can present with psychotic features. The temporal lobe is broken down into two parts, the neocortex (including the superior, middle, and inferior temporal gyri), and the mesial temporal lobe (which includes the hippocampus and amygdala). The function of these structures are diverse and include processing auditory stimuli, memory formation, learning, decision making and emotional regulation (2). Thus, it is not surprising that studies have demonstrated temporal lobe abnormalities in patients with psychotic symptoms, including those with schizophrenia (3, 4). In a meta-analysis of published case studies of CNS tumors that presented with psychiatric symptoms, 22 out of 88 tumors originated in the temporal lobe. Of those, only 4 of those case reports reported psychotic features induced by the tumor (5). While psychotic symptoms often improve with the removal of the tumor (5), some patients’ symptoms might continue to progress, even after resection of the tumor. As of now, there are no specific treatment guidelines for CNS tumor-induced psychotic disorder, so persistent hallucinations and delusions should follow standard antipsychotic treatments (6).

**Table 1:** Causes of psychotic disorder.

Causes	Specific Examples
Substance induced	cannabis, synthetic cannabinoids, methamphetamines, cocaine, LSD, MDMA, mescaline, withdrawal from alcohol/benzodiazepines
Infectious	HIV, RVR
Nutritional deficiencies	vitamin B12, vitamin D
Endocrine dysfunction	hypothyroidism, hyperthyroidism, Cushing’s syndrome
Autoimmune diseases	systemic lupus erythematosus, multiple sclerosis, NMDA encephalopathy
Genetic disorders	Prader-Willi syndrome, Huntington’s disease, velocardiofacial syndrome, Fahr’s disease
Seizure	temporal lobe epilepsy, focal seizure, nonconvulsive seizure, postictal psychosis
Malignancies	Tumors of cortical, pituitary, pineal, temporal, and limbic structures (most commonly associated)
TBI	Subdural hematoma
Other	Wilson’s disease, narcolepsy, metachromatic leukodystrophy

This table illustrates different etiologies which can cause psychosis (7).

It is also important to understand the etiology of primary psychotic disorders, including schizophrenia. The average age of onset of schizophrenia is found to be 10 to 25 in males and 25 to 35 in females (8). Large population studies found that the incidence of the first episode of a psychotic disorder in people aged 30 to 59 was half as much as those aged 15 to 29 (9). This means that though people are twice as likely to develop the first incident of psychosis by their 20s, up to 1/3 of patients will theoretically develop psychotic disorders in their 30s to 50s. While age can serve as a useful indicator at the far ends of the age spectrum, it falls short as a reliable filter for uncovering secondary causes of psychotic disorder.

Substance use disorder is often a confounding factor regarding understanding the etiology of psychosis. Methamphetamine can induce psychosis by causing stimulated release of dopamine, norepinephrine and serotonin while also acting as a reuptake inhibitor (10). The pharmacological effect of this drug can cause auditory and visual hallucinations, disorganized speech, ideas of reference and other delusions (11). One meta-analysis found the prevalence of methamphetamine-induced psychotic disorder to be 36.5% in those that use methamphetamines (12). These numbers are even more staggering when it is estimated that 310,000 people use methamphetamines daily in the United States (13). Pharmacokinetically, the half-life of methamphetamines is approximately 10 hours, meaning it takes 36-48 hours for blood levels to be undetectable (14). Pharmacological considerations can be used to attribute methamphetamine-induced psychotic disorder vs. another etiology. Though our patient had a positive urine toxicology screen for amphetamines, his delusions remained well past a 48-hour window of stimulant withdrawal, which makes the etiology of his psychotic symptoms more plausibly to be attributed to the direct effects of the brain tumor. This does not rule out other causes such as a new primary diagnosis of a psychotic disorder.

Our patient’s psychotic symptoms initially improved after resection and radiation therapy but recurred. This was potentially due to the repeated consumption of amphetamines and/or due to microscopic structural damage induced by the tumor and its resection, though there was no sign of tumor recurrence on subsequent neuroimaging.

Although there are currently no specific treatments of psychotic disorder secondary to temporal lobe tumors or stimulants, an effective medication for treating positive symptoms in psychotic disorders is risperidone. One meta-analysis found risperidone to have one of the largest standard mean difference in improvement of positive symptoms of psychosis at -0.61 (-0.68 to -0.54), only second to that of the more expensive amisulpride, -0.69 (-0.86 to -0.52) (15). These medications can be transitioned to the long-acting injectable form of risperidone or the pharmacologically similar paliperidone, which also boasts a strong effect on positive symptoms, with a standard mean difference of -0.53 (-0.65 to -0.42).

In conclusion, a new presentation of psychotic illness must always start with a broad differential and require a thorough evaluation, including history, physical, neuroimaging, and laboratory studies. If a tumor is found on neuroimaging, prompt neurosurgery consultation is needed. Surgical resection may avoid potential complications associated with mass effect. However, even with the removal of the tumor, patients can still experience persistence or recurrence of psychotic symptoms. Antipsychotics can be used to manage residual psychotic symptoms attributable to CNS tumors. This research highlights the time course and treatment of a rare case of psychosis in a person who had a temporal lobe tumor. More research and case reports are needed to highlight the optimal treatment for patients with this presentation of psychosis.

## Data Availability

The original contributions presented in the study are included in the article/supplementary material. Further inquiries can be directed to the corresponding author.
